# Flavan-3-ols consumption and cancer risk: a meta-analysis of epidemiologic studies

**DOI:** 10.18632/oncotarget.12017

**Published:** 2016-09-14

**Authors:** Lei Lei, Ying Yang, Hongjuan He, Erfei Chen, Le Du, Jing Dong, Jin Yang

**Affiliations:** ^1^ Key Laboratory of Resources Biology and Biotechnology in Western China, Ministry of Education, College of Life Science, Northwest University, Xi'an 710069, China; ^2^ Institute of Preventive Genomic Medicine, Xi'an 710069, China

**Keywords:** meta-analysis, flavan-3-ols, cancer, colorectal cancer, breast cancer

## Abstract

Although numerous *in vitro* studies and animal model data have suggested that flavan-3-ols, the most common subclass of flavonoids in the diet, may exert protective effects against cancer, epidemiologic studies have reported inconclusive results for the association between flavan-3-ols intake and cancer risk. Therefore, we conducted this meta-analysis of epidemiologic studies to investigate the preventive effects of flavan-3-ols on various types of cancers. A total of 43 epidemiologic studies, consisting of 25 case-control and 18 prospective cohort studies, were included. A significant inverse association was shown between flavan-3-ols intake and the risk of overall cancer (relative risk (RR) 0.935, 95%CI: 0.891-0.981). When cancer types were separately analyzed, a statistically significant protective effect of flavan-3-ols consumption was observed in rectal cancer (RR 0.838, 95%CI: 0.733-0.958), oropharyngeal and laryngeal cancer (RR 0.759, 95%CI: 0.581-0.993), breast (RR 0.885, 95%CI: 0.790-0.991) in case-control studies and stomach cancer in women (RR 0.633, 95%CI: 0.468-0.858). Our analysis indicates the potential benefits of flavan-3-ols in cancer prevention.

## INTRODUCTION

Dietary flavonoids are a diverse range of polyphenols that are ubiquitously found in many commonly consumed vegetables, fruits, food grains, herbal remedies, beverages, and dietary supplements. Most total dietary flavonoids are flavan-3-ols (83.5%), followed by flavanones, flavonols, anthocyanidins, flavones and isoflavones, as estimated based on the United States Department of Agriculture (USDA) database [1] and the mean daily flavonoid intake reported for adults in the United States [2]. Flavan-3-ols include (+)-catechin, (+)-gallocatechin, (−)-epicatechin, (−)-epigallocatechin, (−)-epicatechin 3-gallate, (−)-epigallocatechin 3-gallate, theaflavin, theaflavin 3-gallate, theaflavin 3′-gallate, theaflavin 3, 3′-digallate and thearubigins [1], which are abundant in tea, wine, apples and cocoa. An extensive literature indicates that flavan-3-ols exhibit a range of biological activities both *in vitro* and *in vivo* by acting as an antioxidant, cardio-preventive, anticarcinogenic, anti-microbial, anti-viral, and neuro-protective agent [3].

To date, epidemiological studies have focused primarily on flavan-3-ols and cardiovascular disease, and meta-analyses have suggested a reduced risk of cardiovascular disease associated with flavan-3-ols intake [4, 5]. Furthermore, when focusing on cancer preventive effects, meta-analyses of observational studies have mainly indicated an association between cancer risk and intake of other flavonoids, such as isoflavones [6, 7]. Although numerous *in vitro* and animal model studies have suggested that flavan-3-ols act as anticarcinogens through antioxidant [8, 9] and detoxifying effects [10], modulation of the cell cycle and apoptosis [11-13], stimulation of the immune system [14] and DNA repair [15], and suppression of metastasis [16, 17] and inflammation [17-19], data from human population studies are limited and conflicting [20].

To our knowledge, no quantitative evaluations of the association between flavan-3-ols intake and the risk of overall cancer have been reported. Thus, we performed a quantitative meta-analysis of the currently available epidemiologic studies to estimate the effects of flavan-3-ols consumption on cancer risk.

## RESULTS

### Literature search

**Figure 1:**
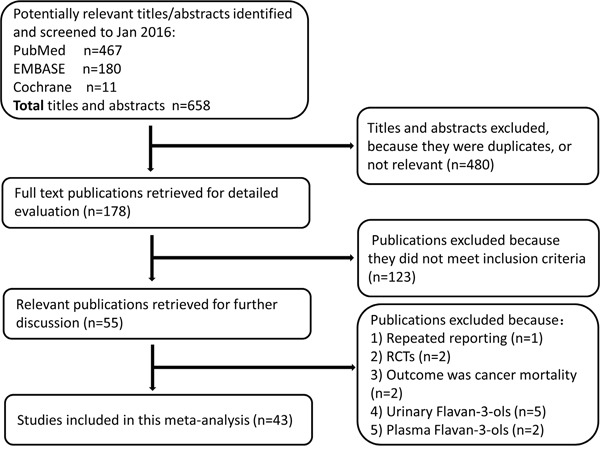
Flow diagram illustrating the reference search and selection in this meta-analysis (RCTs, randomized controlled trials)

We screened 658 titles or abstracts from the 3 databases, of which 178 were reviewed in full. The flow diagram is shown in Figure 1. A total of 43 studies were included in this meta-analysis, and the main reasons for excluding studies from the final review were as follows: 1 study was repeated reporting [21], 2 studies were randomized controlled trials (RCTs) [22, 23], 2 studies had an outcome of cancer mortality [24, 25], 5 studies evaluated urinary flavan-3-ols [26-30], and 2 studies evaluated plasma flavan-3-ols [31, 32].

### Study characteristics and quality assessment

The main characteristics of the included studies are shown in Table 1. The types of study design were as follows: cohort studies (n=18), hospital-based case-control studies (n=18), and population-based case-control studies (n=7). 13 studies were conducted in the Americas (USA, n=11; Mexico, n=1; and Canada, n=1), 29 studies were conducted in Europe (Multiple European countries, n=5; Italy, n=11; Greece, n=4; France, n=1; UK, n=2; Finland, n=2; Netherlands, n=3; and Spain, n=1), and only one study was conducted in Asia (Korea, n=1). Of these studies, 7 evaluated catechins as a major flavan-3-ols [33-39] and others assessed flavan-3-ols according into the USDA databases for the flavonoid content of selected foods [1] (Supplementary Table 1). These studies covered 14 different types of cancer, most of which were adjusted for a wide range of confounders, including age, total energy intake, BMI, physical activity, smoking status, alcohol intake, and total fruit and vegetable consumption. All studies used food-frequency questionnaires to measure flavan-3-ols intake.

**Table 1:** Characteristics of included studies

Study	Region and design	Cases/cohort or controls	Follow-up (years)	Flavonoid type	Intake comparison	Cancer site	RR/OR/HR (95% CI)	Adjustments
Arts,2002 [33]	USA; Cohort	5,038/34,651	13	Total catechin: ((+)-catechin; (−)-epicatechin; (+)-gallocatechin; (−)-epigallocatechin; (−)-epicatechin gallate; (−)-epigallocatechin gallate)	75.1 VS 3.6 mg/d	Any cancer combined	0.97 (0.88–1.06)	age, total energy intake, body mass index, waist-to-hip ratio, physical activity, pack-years of smoking, smoking status, number of years since quit smoking, alcohol intake, and total fruit and vegetable consumption
					24.7 VS 3.6 mg/d	Upper digestive tract	0.71 (0.46-1.11)	
					75.1 VS 3.6 mg/d	Colon	1.10 (0.85–1.44)	
					24.7 VS 3.6 mg/d	Rectum	0.55 (0.32–0.95)	
					24.7 VS 3.6 mg/d	Pancreas	0.74 (0.46–1.20)	
					75.1 VS 3.6 mg/d	Bronchus and lung	0.94 (0.72–1.23)	
					24.7 VS 3.6 mg/d	Hematopoietic	0.65 (0.43–0.98)	
					75.1 VS 3.6 mg/d	Breast	1.04 (0.84–1.28)	
					24.7 VS 3.6 mg/d	Uterus	1.00 (0.73–1.36)	
					24.7 VS 3.6 mg/d	Ovary	0.73 (0.44–1.24)	
					24.7 VS 3.6 mg/d	Kidney and renal pelvis	0.73 (0.40–1.32)	
					14.8 VS 3.6 mg/d	Bladder	1.12 (0.65–1.93)	
					24.7 VS 3.6 mg/d	Non-Hodgkin's lymphoma	1.26 (0.87–1.85)	
Zamora-Ros,2014 [69]	Europe; Cohort	1,575/477,312	11	Flavan-3-ol monomers	>376.0 VS <19.3 mg/d	Bladder	0.90 (0.73–1.11)	sex, age (1 year) and centre and adjusted for energy, smoking intensity, BMI, physical activity, alcohol intake and highest educational level.
Bosetti,2005 [70]	Italy; Case control (hospital based)	2,569/2,588	-	Flavan-3-ols	>79.7 VS 18.1 mg/d	Breast total	0.86 (0.71-1.05)	
					>18.1 VS 18.1 mg/d	Breast premenopause	0.94 (0.85-1.05)	age, study center, education, parity, alcohol consumption, and non-alcohol energy intake
						Breast postmenopause	0.92 (0.84-1.00)	
Fink,2007 [71]	USA; Case control (population based)	1,434 /1,440	-	Flavan-3-ols	≥268.0 VS ≤6.5 mg/d	Breast total	0.85 (0.67-1.08)	age and energy intake
					≥264.2 VS ≤5.1 mg/d	Breast premenopause	1.21 (0.78-1.86)	
					≥278.0 VS ≤7.6 mg/d	Breast postmenopause	0.74 (0.55-0.99)	
Peterson,2003 [72]	Greece; Case control (hospital based)	820 / 1,548	-	Flavan-3-ols	Per 1 s.d. increment	Breast	0.93 (0.78 −1.11)	age, place of birth, parity, age at first pregnancy, age at menarche, menopausal status, body mass index, total energy intake, alcohol consumption, fruit and vegetable consumption and mutually between flavonoid categories
Sanchez,2009 [73]	Mexico; Case control (hospital based)	141/141	-	Flavan-3-ols	10.6–45.9 VS 0.2–5.9 mg/d	Breast total	0.80 (0.38-1.70)	age, total energy consumption, lifetime lactation (months) and menopausal stage
						Breast premenopause	1.22 (0.48-3.08)	
						Breast postmenopause	0.63 (0.25-1.62)	
						Breast higher alcohol	2.28 (1.19–4.36)	age, BMI, intervention group, alcohol intake, number of dietary records, energy intake, height, physical activity, smoking status, educational level, family history of breast cancer, menopausal status, use of hormonal treatment for menopause, and number of children
Touvier,2013 [34]	France; Cohort	152/4,141	12.6	catechins	151.5 VS 61.2 mg/d			
						Breast low alcohol	0.48 (0.22–1.05)	
Wang,2014 a [74]	USA; Cohort	2116 /56,630	8.5	Flavan-3-ols	>36.7–410 VS ≤9.0 mg/d	Breast	0.98 (0.86–1.12)	age, family history of breast cancer, history of breast cyst, weight gain since age 18 y, education, combination of parity and age at first live birth, ethanol consumption, smoking history, total energy intake in quintile, age at menopause and history of hormone replacement therapy
Zamora-Ros,2013 a [75]	Europe; Cohort	11,576/334,850	11.5	Flavan-3-ol monomers	>379.8 VS <18.2 mg/d	Breast total	1.01 (0.93–1.09)	age, menopausal status, weight, height, smoking status, educational level, physical activity, age at menarche, age at first full-term birth, ever use of contraceptive, use of hormones, age at menopause, energy intake, alcohol intake and fibre intake
						Breast premenopause	0.96 (0.82–1.13)	
						Breast postmenopause	1.00 (0.90–1.11)	
Kyle,2010 [76]	UK; Case control (population based)	261/404	-	Flavan-3-ols	>188.81 VS <67.10 mg/d	Colorectum	0.6 (0.4 - 1.0)	energy, age at diagnosis, family history, non-steroidal anti-inflammatory drugs, aspirin, Mn, riboflavin, vitamin C, folate
						Colon	0.5 (0.3 - 1.0)	
						Rectum	0.7 (0.4 - 1.4)	
Mursu,2008 [77]	Finland; Cohort	Lung 62, prostate 138, and colorectum 55 /2590	16.2	Flavan-3-ols	Quartile 4 VS 1	Colorectum	1.37 (0.65–2.89)	age, examination years, BMI, smoking status, pack-years of smoking, physical activity, intakes of alcohol, total fat, saturated fat, energy adjusted intake of fiber, vitamin C and E
						Lung	0.24 (0.09–0.64)	
						Prostate	1.13 (0.70–1.82)	
Nimptsch,2016 [44]	USA cohort	Male: 1061/42478 Female: 1458/76364	26	Flavan-3-ols	Male: 128±88 VS 11.9±7.2 mg/d Female: 149.5±95.1 VS 9.3±5.1 mg/d	Colorectum	1.07 (0.95, 1.21)	age, pack-years of smoking before age 30 y, history of colorectal cancer in a parent or sibling, history of endoscopy, regular aspirin use, BMI, physical activity, alcohol consumption, total calories, energy-adjusted total vitamin D intake, total calcium intake, red meat intake, and processed meat intake
						Colorectum male	1.12 (0.92, 1.36)	
						Colorectum female	1.04 (0.88, 1.23)	
						Colon	1.09 (0.93, 1.27)	
						Colon male	1.20 (0.96, 1.50)	
						Colon female	1.02 (0.85, 1.23)	
						Rectum	1.02 (0.78, 1.34)	
						Rectum male	0.90 (0.60, 1.35)	
						Rectum female	1.13 (0.79, 1.63)	
Rossi,2006 [78]	Italy; Case control (hospital based)	1953 /4154	-	Flavan-3-ols	>88.5 VS <20.8 mg/d	Colorectum	0.98 (0.82-1.18)	age, sex, study center, family history, education, alcohol consumption, body mass index, occupational physical activity, and energy intake
						Colon	1.12 (0.90-1.39)	
						Rectum	0.81 (0.62-1.06)	
Simons,2009 [35]	Netherland; Case-cohort	Male: 1,444/2,191 Female: 1,041/2,247	13.3	Total catechin ((+)-catechin; (−)-epicatechin; (+)-gallocatechin; (−)-epigallocatechin; (−)-epicatechin gallate; (−)-epigallocatechin gallate)	84.3–290.1 VS <24.2 mg/d	Colorectum male	0.99 (0.77–1.25)	age, family history of colorectal cancer, smoking status, alcohol intake, occupational physical activity at longest held job, BMI and processed meat intake
						Colon male	1.13 (0.86–1.48)	
						Rectum male	0.80 (0.56–1.14)	
					95.9–287.3 VS <36.2 mg/d	Colorectum female	0.79 (0.61–1.02)	
						Colon female	0.82 (0.62–1.09)	
						Rectum female	0.80 (0.48–1.33)	
Theodoratou, 2007 [79]	Scotland; Case control (population based)	1,456/1,456	-	Flavan-3-ols	>162.1 VS 0-42.6 mg/d	Colorectum	0.81 (0.65-1.01)	age, gender, family history, total energy intake, total fiber intake, alcohol intake, NSAID intake, smoking, BMI, and physical activity
						Colon	0.79 (0.59-1.07)	
						Rectum	0.87 (0.62-1.24)	
Zamora-Ros, 2013 b [80]	Spain; Case control (hospital based)	424 /401	-	Flavan-3-ols	>12.9 VS <4.9 (mg/1,000 kcal day)	Colorectum	0.79 (0.49 - 1.28)	sex, age, BMI, energy intake, alcohol, fiber intake, red and processed meat intake, tobacco consumption, physical activity, regular drugs and family history
						Colon	0.78 (0.45–1.34)	
						Rectum	0.78 (0.39–1.54)	
Bobe,2009 [81]	USA; Case control (population based)	161EAC 114ESCC / 678 white 218ESCC /557 black	-	Flavan-3-ols	>60.6 VS <10.3 mg/1,000 kcal >49.4 VS <6.17 mg/1,000 kcal	Esophagus White-EAC	1.22 (0.60–2.49)	smoking, geographical area, age, BMI, hot tea, hard liquor and beer, moonshine consumption (only for black men), red wine, white wine consumption (except for ESCC in white men), caloric intake, education (only for black men) and income
						Esophagus White-ESCC	0.95 (0.36–2.52)	
						Esophagus Black-ESCC	0.78 (0.36–1.68)	
Petrick,2015 [82]	USA; Case control (population based)	EAC 274, ESCC 191, GCA 248, OGA 341 / 622	-	Flavan-3-ols	≥130.7 VS 0–10.29 mg/d	Esophagus EAC	1.02 (0.69-1.51)	age, sex, race, geographic center, cigarette smoking and dietary energy intake
						Esophagus ESCC	0.98 (0.60-1.59)	
						Stomach GCA	1.17 (0.77-1.78)	
						Stomach OGA	1.30 (0.88-1.92)	
Rossi,2007 a [83]	Italy; Case control (hospital based)	304/743	-	Flavan-3-ols	>109.1 VS <32.6 mg/d	Esophagus ESCC	1.06 (0.58–1.94)	age, sex, study center, education, alcohol consumption, tobacco smoking, BMI and energy intake
Vermeulen, 2013 [84]	Europe; Cohort	341 / 477,312	11	Flavan-3-ol monomers	Quartile 4 VS 1	Esophagus	0.86 (0.58-1.27)	age, sex, energy intake, BMI, smoking intensity, educational level, physical activity, alcohol, red and processed meat intake, fiber, vitamin C and carotenoids
Lagiou,2004 a [85]	Greece; Case control (hospital based)	110/100	-	Flavan-3-ols	Per 1 s.d. increment	Stomach	1.04 (0.68–1.58)	age, gender, place of birth, BMI, height, years of education, smoking habits and duration of smoking, alcohol consumption, total energy intake, fruit and vegetable consumption
Rossi,2010 [86]	Italy; Case control (hospital based)	230/547	-	Flavan-3-ols	>79.2 VS <21.6 mg/d	Stomach	0.75 (0.45–1.23)	sex, age, education, year of interview, BMI, tobacco smoking, and total energy intake
Woo,2014 [87]	Korea; Case control (hospital based)	334/334	-	Flavan-3-ols	39.7 VS 3.2 mg/d	Stomach	0.73 (0.45–1.18)	total energy intake, H. pylori, age, sex, education, smoking status, alcohol consumption, BMI, physical activity, consumption of pickled vegetable and red and processed meat, fruits and vegetable consumption
						Stomach male	0.78 (0.41–1.49)	
						Stomach female	0.65 (0.27–1.57)	
Zamora-Ros, 2012 [88]	Europe; Cohort	683/ 477,312	11	Flavan-3-ol monomers	>199.9 VS <26.1 mg/d >241.6 VS <21.1 mg/d	Stomach male	0.98 (0.68, 1.40)	center, sex, age, educational level, smoking status, physical activity, BMI, alcohol and energy intake, daily consumption of fruit, vegetables, and red and processed meat
						Stomach female	0.55 (0.34, 0.88)	
Rossi, 2007 b [89]	Italy; Case control (hospital based)	805/2,081	-	Flavan-3-ols	>99.6 VS <23.3 mg/d	Oropharynx	0.84 (0.60-1.18)	sex, age, study center, smoking, alcohol drinking, education, BMI, and non–alcohol energy intake
Garavello, 2007 [90]	Italy; Case control (hospital based)	460/1,088	-	Flavan-3-ols	>110.4 VS <31.2 mg/d	Larynx	0.64 (0.41–0.99)	age, sex, study center, education, alcohol consumption, smoking, BMI, occupational physical activity and non-alcohol energy intake
Lagiou,2008 [91]	Greece; Case control (hospital based)	250 HBV and/or HCV positive, 83 HBV and HCV negative /360	-	Flavan-3-ols	>66.3 VS <25.3 mg/d	Liver virus positive	1.17 (0.65–2.12)	gender, age, education, tobacco smoking, and total energy intake
						Liver virus negative	1.14 (0.50–2.58)	
Zamora-Ros, 2013 c [92]	Europe; Cohort	191/477,206	11	Flavan-3-ol monomers	>134.8 VS <28.2 mg/d	Liver	0.65 (0.39-1.07)	center, sex, age, total energy, education, smoke intensity, alcohol lifetime and alcohol baseline, BMI, self-reported diabetes at baseline, physical activity, fiber and coffee intake
Arts, 2001 [36]	Netherland; Cohort	96 Epithelial cancer, 42 lung cancer /728	10	Total catechin((+)-catechin; (−)-epicatechin; (+)-gallocatechin; (−)-epigallocatechin; (−)-epicatechin gallate; (−)-epigallocatechin gallate)	85.8–355.4 VS 0–49.0 mg/d	Epithelial cancer combined	0.94 (0.56–1.59)	age, physical activity, total energy intake, alcohol intake, smoking status, pack-years of smoking, BMI, coffee, fiber, vitamin C, vitamin E, beta-carotene
						Lung	0.92 (0.41–2.07)	
Christensen, 2012 [93]	Canada; Case control (population based)	1,061/1,425	-	Flavan-3-ols	Male: >271.0 VS <12.6 mg/d Female: >249.1 VS <8.7 mg/d	Lung	1.06 (0.81–1.39)	age, sex, number of school years, mean census tract family income, ethnic group, respondent status, comprehensive smoking indicator, occupational exposure to carcinogens, BMI, number of alcoholic drinks/day and total energy intake
						Lung smokers	1.08 (0.79–1.49)	
						Lung nonsmokers	1.01 (0.56–1.81)	
						Lung male	1.18 (0.84–1.67)	
						Lung female	0.85 (0.53–1.35)	
Cui,2008 [37]	USA; Case control (population based)	558/837	-	Epicatechin	≥9 VS <3 mg/d	Lung	0.66 (0.46–0.94)	age, sex, race-ethnicity, years of schooling, smoking status, pack-years of tobacco smoking, and daily energy intake
						Lung smokers	0.61 (0.40–0.93)	
						Lung nonsmokers	0.81 (0.40–1.60)	
				Catechin	≥3 VS <1 mg/d	Lung	0.54 (0.38–0.76)	
						Lung smokers	0.44 (0.29–0.66)	
						Lung nonsmokers	0.77 (0.39–1.50)	
Cutler,2008 [94]	USA; Cohort	All cancer 7,534, lung cancer 849 / 34,708	19	Flavan-3-ols	134.8–1051.6 VS <0.001–6.7 mg/d	Any cancer combined	0.97 (0.90–1.05)	age, energy, education level, race, BMI, multivitamin use, smoking history and pack years, activity level
						Lung smokers	1.07 (0.85–1.36)	
						Lung nonsmokers	1.04 (0.54–2.02)	
Lagiou,2004 b [95]	Greece; Case control (hospital based)	154/145	-	Flavan-3-ols	Per 1 s.d. increment	Lung	1.02 (0.70–1.49)	age, total energy intake, smoking status, pack-years, fruit and vegetable consumption
Rossi,2008 [96]	Italy; Case control (hospital based)	1,031/ 2,411	-	Flavan-3-ols	>77 VS <16.3 mg/d	Ovary	0.89 (0.67–1.17)	age, study center, education, year of interview, parity, oral contraceptive use and family history of ovarian or breast cancer or both in first-degree relatives
Arem,2013 [97]	USA; Cohort	2,379/537,104	10.6	Flavan-3-ols	331.2 VS 8.6 mg/d	Pancreas	1.03 (0.91–1.17)	sex, smoking, diabetes, BMI, alcohol, calories, saturated fat and red meat intake, with age as the underlying time metric
Bobe,2008 [39]	Finland; Cohort	306/27,111	16.1	Flavan-3-ols	>9.75 VS ≤0.90 mg/d	Pancreas	0.92 (0.64-1.31)	age, smoking, total number of cigarettes per day, self-reported history of diabetes mellitus, and energy-adjusted saturated fat intake
Rossi,2012 [98]	Italy; Case control (hospital based)	326/652	-	Flavanols	>97.7 VS ≤23.8 mg/d	Pancreas	0.63 (0.38–1.03)	gender, age, center of study, year of interview, education, history of diabetes, smoking, alcohol drinking and non-alcohol energy intake
Bosetti,2006 [99]	Italy; Case control (hospital based)	1,294/1,451	-	Flavan-3-ols	≥102.1 VS <29.9 mg/d	Prostate	1.30 (1.01–1.69)	age, study center, education, BMI, family history of prostate cancer and total calorie intake.
Geybels, 2013 [38]	Netherlands; Cohort	3,362/58,279	18	Total catechin: ((+)-catechin; (−)-epicatechin; (+)-gallocatechin; (−)-epigallocatechin; (−)-epicatechin gallate; (−)-epigallocatechin gallate)	98.7 VS 14.5 mg/d	Prostate	0.97 (0.82-1.15)	age, flavonoid intake, family history of prostate cancer, smoking, nonoccupational physical activity, BMI, height, diabetes, education and intake of energy, alcohol, calcium, lycopene, vitamin E, red meat and coffee
Wang,2014 b [100]	USA; Cohort	3,974/43,268	7.8	Flavan-3-ols	≥73.9 VS <10.4 mg/d	Prostate	1.18 (1.06-1.30)	age, race, family history of prostate cancer, BMI in 1999, smoking status, aspirin use, total energy intake, history of prostate-specific antigen screening, and diabetes
Bosetti,2007 [101]	Italy; Case control (hospital based)	767/1,534	-	Flavan-3-ols	≥90.6 VS <21.3 mg/d	Kidney	0.77 (0.56-1.06)	sex, age, study center, period of interview, education, smoking, alcohol drinking, BMI, occupational physical activity, family history of kidney cancer, and total energy intake
Xiao,2014 [102]	USA; Cohort	586/491,840	9	Flavan-3-ols	161.89–7205.21 VS 0.00–17.16 mg/d	Thyroid	0.66 (0.51, 0.85)	sex, total caloric intake, smoking status, education level, alcohol intake, race, BMI, family history of cancer
Rossi,2013 [103]	Italy; Case control (hospital based) control (hospital based)	454/908	-	Flavanols	IV vs I–III quartile category	Uterus	0.99 (0.74–1.32)	age, year of interview, education, BMI, history of diabetes, age at menarche, menopausal status/age at menopausal status, parity, oral contraceptive use, and hormone-replacement therapy use

The quality of the included studies varied, as assessed using the Newcastle-Ottawa Scale, as shown in Tables 2 and 3. The ranges of quality scores were 8-10 in the cohort studies and 6-9 in the case-control studies. Therefore, all of the cohort studies (n=18) and 84% of the case-control studies (n=21) were high-quality studies (studies with a score≥7).

**Table 2:** Quality assessment scale score for cohort studies included in the meta-analysis^1^

First author, year of publication (reference)	Representativeness of the exposed cohort	Selection of the unexposed cohort	Ascertainment of exposure	Outcome of interest not present at start of study	Control for important factor or additional factor^2^	Outcome assessment	Follow-up long enough for outcomes to occur^3^	Adequacy of follow-up of cohorts^4^	Data analysis that used an energy-adjusted residual or nutrient-density model	Total quality scores
Arts,2002 [33]	☆	☆	☆	☆	☆☆	☆	☆	-	☆	9
Zamora-Ros,2014 [69]	☆	☆	☆	☆	☆☆	☆	☆	-	☆	9
Touvier,2013 [34]	☆	☆	☆	☆	☆☆	☆	☆	-	☆	9
Wang,2014 a [74]	☆	☆	☆	☆	☆☆	☆	☆	☆	☆	10
Zamora-Ros,2013 a [75]	☆	☆	☆	☆	☆☆	☆	☆	-	☆	9
Mursu,2008 [77]	☆	☆	-	☆	☆☆	☆	☆	☆	☆	9
Nimptsch,2016 [44]	-	☆	☆	☆	☆☆	☆	☆	☆	-	8
Simons,2009 [35]	-	☆	☆	☆	☆☆	☆	☆	☆	-	8
Vermeulen,2013 [84]	☆	☆	☆	☆	☆☆	☆	☆	-	☆	9
Zamora-Ros,2012 [88]	☆	☆	☆	☆	☆☆	☆	☆	-	☆	9
Zamora-Ros,2013 c [92]	☆	☆	☆	☆	☆☆	☆	☆	-	☆	9
Arts,2001 [36]	☆	☆	☆	☆	☆☆	☆	☆	☆	☆	10
Cutler,2008 [94]	☆	☆	☆	☆	☆☆	-	☆	☆	☆	9
Arem,2013 [97]	☆	☆	☆	☆	☆☆	☆	☆	-	☆	9
Bobe,2008 [39]	-	☆	☆	☆	☆☆	☆	☆	-	☆	8
Geybels,2013 [38]	-	☆	☆	☆	☆☆	☆	☆	☆	☆	9
Wang,2014 b [100]	☆	☆	☆	☆	☆☆	☆	-	-	☆	8
Xiao,2014 [102]	☆	☆	☆	☆	☆☆	☆	☆	-	☆	9

1 A study could be awarded a maximum of one star for each item, while two stars for the item Control for important factor or additional factor.

2 A maximum of two stars could be awarded for this item. Studies similar to others in terms of patient characteristics (age, sex, BMI) received one star, whereas studies that controlled for other important confounders such as smoking and drinking received an additional star.

3 A cohort study with a follow-up time >8 y was awarded one star.

4 A cohort study with a follow-up rate >75% was awarded one star.

**Table 3:** Quality assessment scale score for case-control studies included in the meta-analysis^1^

First author, year of publication (reference)	Adequate definition of cases	Representativeness of cases	Selection of control subjects	Definition of control subjects	Control for important factor or additional factor^2^	Exposure assessment	Same method of ascertainment for all subjects	Nonresponse rate^3^	Data analysis that used an energy-adjusted residual or nutrient-density model	Total quality scores
Bosetti,2005 [70]	☆	☆	-	☆	☆☆	-	☆	☆	☆	8
Fink,2007 [71]	☆	☆	☆	-	☆☆	-	☆	-	☆	7
Peterson,2003 [72]	☆	☆	-	-	☆☆	-	☆	-	☆	6
Sanchez,2009 [73]	☆	☆	-	☆	☆☆	-	☆	☆	☆	8
Kyle,2010 [76]	☆	☆	☆	-	☆☆	-	☆	☆	☆	8
Rossi,2006 [78]	☆	☆	-	☆	☆☆	-	☆	☆	☆	8
Theodoratou,2007 [79]	☆	☆	☆	-	☆☆	-	☆	-	☆	7
Zamora-Ros,2013 b [80]	☆	☆	-	☆	☆☆	-	☆	☆	☆	8
Bobe,2009 [81]	☆	☆	☆	☆	☆☆	-	☆	☆	☆	9
Petrick,2015 [82]	☆	☆	☆	-	☆☆	-	☆	-	☆	7
Rossi,2007 a [83]	☆	☆	-	☆	☆☆	-	☆	☆	☆	8
Lagiou,2004 a [85]	☆	☆	-	-	☆☆	-	☆	-	☆	6
Rossi,2010 b [86]	☆	☆	-	☆	☆	-	☆	☆	☆	7
Woo,2014 [87]	☆	☆	-	☆	☆☆	-	☆	-	☆	7
Rossi,2007 b [89]	☆	☆	-	☆	☆☆	-	☆	☆	☆	8
Garavello,2007 [90]	☆	☆	-	☆	☆☆	-	☆	☆	☆	8
Lagiou,2008 [91]	-	☆	-	☆	☆	-	☆	☆	☆	6
Christensen,2012 [93]	☆	☆	☆	-	☆☆	-	☆	-	☆	7
Cui,2008 [37]	☆	☆	☆	☆	☆☆	-	☆	-	☆	8
Lagiou,2004 b [95]	☆	☆	-	-	☆☆	-	☆	-	☆	6

1 A study could be awarded a maximum of one star for each item, while two stars for the item Control for important factor or additional factor.

2 A maximum of two stars could be awarded for this item. Studies similar to others in terms of patient characteristics (age, sex, BMI) received one star, whereas studies that controlled for other important confounders such as smoking and drinking received an additional star.

3 One star was awarded if there was no significant difference in the response rate between control and cases by chi-square test (P> 0.05).

### Overall cancer

**Figure 2:**
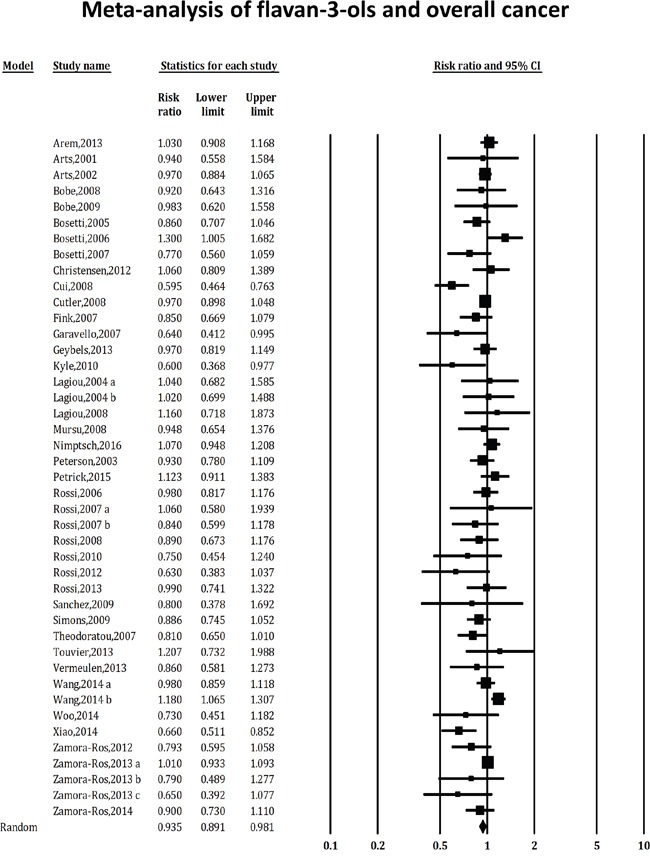
Association of consumption of flavan-3-ols with the risk of overall cancer The square boxes represent study-specific estimates. The size of each box reflects the study's weight in the analysis, and the horizontal lines represent 95% CIs. The diamond indicates the combined relative risk of the analysis using the random-effects model of DerSimonian and Laird.

Our analysis of the 43 studies showed a 6.5% reduction in the risk of overall cancer with high flavan-3-ols consumption (RR 0.935, 95%CI: 0.891-0.981), as shown in Figure 2. Based on Begg's test, there was no indication of publication bias (P=0.07). However, the asymmetry of Egger's funnel plot suggested a possible absence of negative studies (P=0.003). According to the trim and fill analysis, 12 such studies were filled in to the right of the mean to make the funnel symmetrical, and the adjusted estimated effect was RR 0.987 (95%CI: 0.936-1.040) based on the random-effects model. In sensitivity analyses, each study was excluded one by one to determine its influence on the summary risk estimate. The RR of the remaining studies ranged from 0.928 (95%CI: 0.882-0.977) to 0.952 (95%CI: 0.911-0.994) by omission of Zamora-Ros, 2013a and Cui, 2008 studies, respectively, without great fluctuation. Substantial heterogeneity was observed in this analysis (Q=79.002, P=0.000, I^2^=46.837%), which confirmed that flavan-3-ols have different effects on cancer risk, depending on the cancer site.

### Colorectal cancer

**Figure 3:**
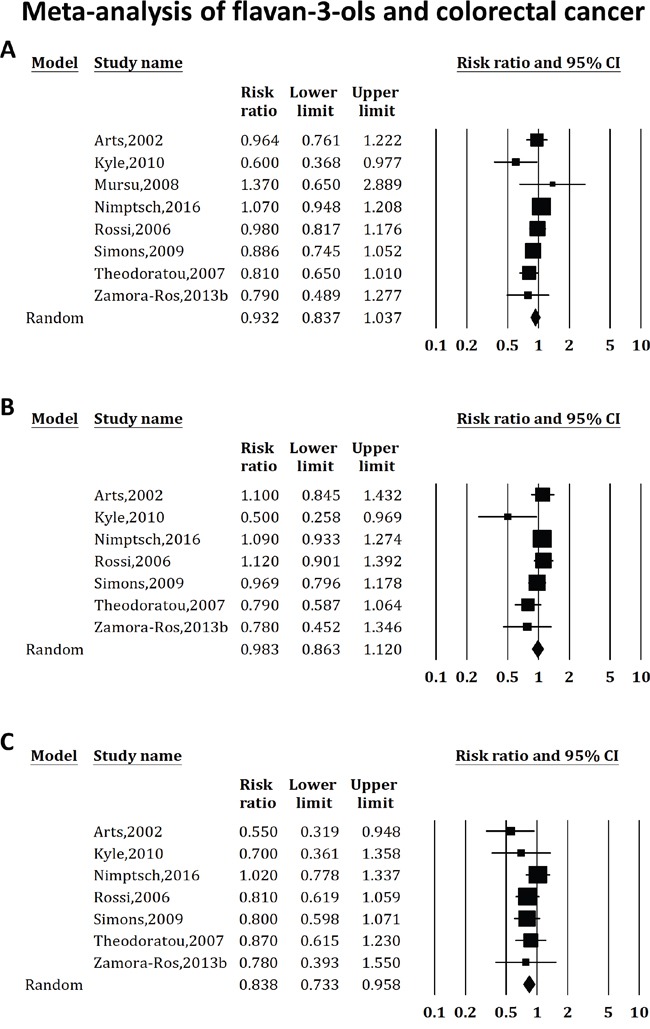
Effect of flavan-3-ols intake on the risk of colorectal (A), colon (B) and rectal (C) cancer Studies were combined using the random-effects model of DerSimonian and Laird. **(A)** The combined relative risk of colorectal cancer was 0.932 (95%CI: 0.837-1.037, P for heterogeneity = 0.125, I^2^ = 38.251%). **(B)** The combined relative risk of colon cancer was 0.983 (95%CI: 0.863-1.120; P for heterogeneity = 0.117, I^2^ = 41.042%). **(C)** The combined relative risk of rectal cancer was 0.838 (95%CI: 0.733-0.958; P for heterogeneity = 0.562, I^2^= 0%).

As shown in Figure 3, we identified 8 studies of flavan-3-ols intake and colorectal cancer risk, which included 3 cohort studies, 1 case-cohort study, 2 population-based case-control studies and 2 hospital-based case-control studies. The data suggested that there was no significant association between flavan-3-ols and colorectal cancer risk (RR 0.932, 95%CI: 0.837-1.037, P for heterogeneity = 0.125, I^2^= 38.251%). No publication bias was detected with either Begg's test (P=0.536) or Egger's test (P=0.239).

When the analysis was stratified by cancer type, 7 studies suggested that flavan-3-ols were not protective in colon cancer (RR 0.983, 95%CI: 0.863-1.120; P for heterogeneity = 0.117, I^2^= 41.042%), and 7 studies revealed a significant association between flavan-3-ols and rectal cancer (RR 0.838, 95%CI: 0.733-0.958; P for heterogeneity = 0.562, I^2^= 0%). When the analysis was stratified by study design, an association with colorectal cancer risk was neither observed in the case-control studies (RR 0.847, 95%CI: 0.771-1.010; 4 studies, P for heterogeneity = 0.219, I^2^= 32.298%), nor in the cohort studies (RR 0.995, 95%CI: 0.888-1.115; 4 studies, P for heterogeneity = 0.275, I^2^= 22.639%). No publication bias was detected in these analyses.

### Subgroup analyses

Table 4 shows the summary estimates of the effects of flavan-3-ols intake on cancer risk by cancer type. When the analysis was stratified by study region, the association between flavan-3-ols intake and cancer risk was observed in Europe (RR 0.926, 95%CI: 0.880-0.974; 29 studies, P for heterogeneity = 0.329, I^2^= 8.894%), but not in Americas (RR 0.961, 95%CI: 0.883-1.046; 13 studies, P for heterogeneity = 0.000, I^2^= 72.020%) or Asia (RR 0.73, 95%CI: 0.45-1.18; 1 study). When the analysis was stratified by study design, protective effects of flavan-3-ols intake on breast cancer were observed in the case-control studies (RR 0.885, 95%CI: 0.790-0.991; 4 studies, P for heterogeneity = 0.904, I^2^= 0%) but not in the cohort studies (RR 1.009, 95%CI: 0.946-1.075; 4 studies, P for heterogeneity = 0.859, I^2^= 0%). When the analysis was stratified by sex, flavan-3-ols consumption significantly reduced gastric cancer risk in women (RR 0.571, 95%CI: 0.376-0.868; 2 studies, P for heterogeneity = 0.743, I^2^= 0%), but not in men (RR 0.928, 95%CI: 0.677-1.272; 2 studies, P for heterogeneity = 0.545, I^2^= 0%). We also found a significant effect of flavan-3-ols intake on reducing the risk of rectal cancer (RR 0.838, 95%CI: 0.733-0.958; 7 studies, P for heterogeneity = 0.562, I^2^= 0%), oropharyngeal and laryngeal cancer (RR 0.759, 95%CI: 0.581-0.993; 2 studies, P for heterogeneity = 0.337, I^2^= 0%), and thyroid cancer (RR 0.66, 95%CI: 0.51-0.85; 1 study), whereas no evidence of a preventive effect was observed for bladder, breast, colon, esophageal, gastric, liver, lung, ovarian, pancreatic, prostate, renal, or uterine cancer.

**Table 4:** Meta-analysis of flavan-3-ols consumption and cancer risk

Cancer site	No. of studies	RR (95% CI)	Heterogeneity test			References
			Q	P	I^2^(%)	
Overall cancer	43	**0.935 (0.891-0.981)**	79.002	0.000	46.837	
Americas	13	0.961 (0.883-1.046)	42.888	0.000	72.020	[33, 37, 44, 71, 73, 74, 81, 82, 93, 94, 97, 100, 102]
Europe	29	**0.926 (0.880-0.974)**	30.733	0.329	8.894	[34-36, 38, 39, 69, 70, 72, 75-80, 83-86, 88-92, 95, 96, 98, 99, 101, 103]
Asia	1	0.73 (0.45-1.18)				[87]
Bladder	2	0.926 (0.761-1.126)	0.54	0.462	0	[33, 69]
Breast	8	0.977 (0.924-1.033)	5.213	0.634	0	[33, 34, 70-75]
premenopause	4	0.957 (0.878-1.043)	1.494	0.684	0	[70, 71, 73, 75]
postmenopause	5	0.944 (0.865-1.031)	5.656	0.226	29.273	[33, 70, 71, 73, 75]
cohort	4	1.009 (0.946-1.075)	0.76	0.859	0	[33, 34, 74, 75]
Case-control	4	**0.885 (0.790-0.991)**	0.566	0.904	0	[70-73]
Colorectum	8	0.932 (0.837-1.037)	11.336	0.125	38.251	[33, 35, 44, 76-80]
colon	7	0.983 (0.863-1.120)	10.177	0.117	41.042	[33, 35, 44, 76, 78-80]
rectum	7	**0.838 (0.733-0.958)**	4.857	0.562	0	[33, 35, 44, 76, 78-80]
cohort	4	0.995 (0.888-1.115)	3.878	0.275	22.639	[33, 35, 44, 77]
Case-control	4	0.847 (0.711-1.010)	4.431	0.219	32.298	[76, 78-80]
Esophagus	4	0.966 (0.790-1.181)	1.217	0.976	0	[81-84]
ESCC	3	0.960 (0.696-1.323)	0.39	0.942	0	[81-83]
EAC	2	1.063 (0.755-1.499)	0.187	0.666	0	[81, 82]
Stomach	6	0.887 (0.724-1.086)	8.313	0.14	39.851	[33, 82, 85-88]
male	2	0.928 (0.677-1.272)	0.366	0.545	0	[87, 88]
female	3	**0.633 (0.468-0.858)**	0.6	0.741	0	[33, 87, 88]
Oropharynx and Larynx	2	**0.759 (0.581-0.993)**	0.92	0.337	0	[89, 90]
Liver	2	0.881 (0.622-1.247)	2.659	0.265	24.779	[91, 92]
Lung	7	0.854 (0.667-1.094)	21.753	0.001	72.417	[33, 36, 37, 77, 93-95]
smokers	3	0.844 (0.527-1.350)	16.86	0	88.138	[37, 93, 94]
nonsmokers	3	0.910 (0.658-1.259)	0.613	0.736	0	[37, 93, 94]
male	3	0.702 (0.299-1.648)	9.05	0.011	77.9	[36, 77, 93]
female	5	0.873 (0.685-1.112)	13.21	0.01	69.721	[33, 37, 93-95]
cohort	4	0.856 (0.599-1.223)	8.564	0.036	64.972	[33, 36, 77, 94]
Case-control	3	0.854 (0.572-1.275)	11.128	0.004	82.027	[37, 93, 95]
Ovary	2	0.851 (0.666-1.088)	0.436	0.509	0	[33, 96]
Pancreas	4	0.895 (0.725-1.104)	5.052	0.168	40.618	[33, 39, 97, 98]
Prostate	4	1.128 (0.994-1.280)	4.936	0.177	39.217	[38, 77, 99, 100]
Kidney	2	0.761 (0.574-1.008)	0.024	0.877	0	[33, 101]
Thyroid	1	**0.66 (0.51-0.85)**				[102]
Uterus	2	0.995 (0.805-1.229)	0.002	0.963	0	[33, 103]

## DISCUSSION

A total of 43 epidemiologic studies on the effects of flavan-3-ols on cancer risk were identified in this review. To our knowledge, this report is the first quantitative meta-analysis to show an association between flavan-3-ols intake and the risk of overall cancer. Our data suggested that high flavan-3-ols consumption was associated with a 6.5% reduction in the risk of overall cancer. The substantial heterogeneity in the analysis confirmed that the effects of flavan-3-ols differed by cancer site. In particular, flavan-3-ols intake significantly reduced the risk of rectal cancer, oropharyngeal and laryngeal cancer, and thyroid cancer. Reductions in the risk of breast in case-control studies and the risk of gastric cancer in women were also observed. However, no effects on the risk of bladder, colon, esophageal, liver, lung, ovarian, pancreatic, prostate, renal, or uterine cancer were observed. In addition, the association between flavan-3-ols intake and cancer risk was observed in Europe but not in Americas or Asia. The possible explanation could be ethnic differences in food structure and genetic backgrounds.

Although this meta-analysis suggested that there was no significant association between colorectal cancer risk and flavan-3-ols consumption, several mechanisms supported by *in vitro* and animal studies remain biologically plausible. More specifically, *in vitro* studies have shown that flavan-3-ols inhibit cell proliferation and induce apoptosis in colorectal cancer cells [40-42]. In addition, animal studies have shown that catechin, a major flavan-3-ols, is able to decrease the number of intestinal tumors. One meta-analysis found an inverse association between flavan-3-ols consumption and colorectal cancer [43], which was inconsistent with our findings, likely because the analysis did not include the most recent study on the topic [44]. In particular, we found that a significant protective effect of flavan-3-ols was observed in cases of rectal, but not colon cancer. Although colon and rectal cancer are often combined, the differences between colon and rectal cancer in terms of incidence rate [45, 46], molecular characterization [47, 48], and risk factors [49] may explain the distinct effects of flavan-3-ols on different parts of the intestine.

No effect of flavan-3-ols on the risk of breast cancer was identified in the present study, which is similar to the findings of a meta-analysis by Hui et al [50], although that analysis included one study that evaluated urinary excretion of polyphenol metabolites (which we excluded) [28]. We excluded studies that evaluated urinary or plasma flavan-3-ols because urinary excretion of catechin metabolites has shown a weaker correlation with intake [51] and because plasma values vary substantially throughout the day [52]. When the analysis was stratified by study design, a statistically significant association between breast cancer risk and flavan-3-ols intake was observed in case-control studies only. The inconsistent conclusions between the two different study designs may be attributed to the presence of more recall and selection biases in case-control studies. For example, case subjects may have remembered their diet differently after a diagnosis of cancer compared with control subjects. Therefore, recall bias, with a misclassification of subjects, could potentially have led to a spurious association. In addition, the potential for selection bias should be considered because hospital-based control subjects who suffer from other diseases may tend to change their dietary patterns and because population-based control subjects who agree to participate in a study may be more health conscious than the general population.

Separate analyses by sex showed a significant inverse association between flavan-3-ols intake and the risk of stomach cancer in women. A Japanese pooled analysis of six cohort studies showed similar results, with green tea consumption (>5 cups/day) decreasing the risk of stomach cancer in women, but not in men [53]. These gender differences could be partly explained by the regulation of sex hormones, such as estrogens. Moreover, a large prospective study reported that associations were observed between the risk of stomach cancer and age at menopause, years of fertility and years since menopause, indicating that female hormones play a protective role in stomach cancer risk [54]. Furthermore, the risk of stomach cancer was reduced in women undergoing hormone replacement therapy, and the risk increased after treatment with an antiestrogenic agent [55, 56]. Flavan-3-ols such as epigallocatechin gallate (EGCG) and epicatechin gallate (ECG) may inhibit estradiol metabolism and enhance uptake, resulting in a moderate increase in estradiol-induced responses [57]. However, too few publications were included in this subgroup analysis to draw any definite conclusions. Further studies are needed to provide insight into the relationship between sex and flavan-3-ols intake.

Several reviews have reported statistically significant inverse associations between lung cancer risk and total dietary flavonoids intake [58, 59]. However, no significant correlations with flavan-3-ols intake have been found [59, 60]. Similar to the finding of our meta-analysis, no reduced risk of lung cancer was observed in prior estimates of the effects of flavan-3-ols, including in subgroup analyses by sex, smoking status, and study design. The differences in estimated effects between total dietary flavonoids and flavan-3-ols may have been caused by the contribution of other flavonoid compounds, such as quercetin, kaempferol and isoflavones [7, 60]

Similar to all meta-analyses, the present study has several limitations. First, the USDA Database for the Flavonoid Content of Selected Foods, which was the first to estimate flavan-3-ols consumption based on dietary data, was published in 2003 [1], so most of the investigations reviewed here were conducted after publication of this flavonoid database. Consequently, limited numbers of previous studies identifying the effects of flavan-3-ols on cancer risk could be included in the current analysis compared with meta-analyses of other flavonoids [7, 61]. Second, data from a single food-frequency questionnaire may not accurately reflect flavan-3-ols consumption, which would likely lead to non-differential misclassification and an underestimation of the true associations. Furthermore, flavan-3-ols content lacks an accurate biomarker and is influenced by a multitude of factors, such as sunlight, season and food processing [1, 51], thus contributing to inconsistency between studies. Third, because only published studies were included in the present analysis, the analysis may have been affected by publication bias. In fact, based on Egger's test, publication bias was indicated in the analysis of overall cancer. However, the sensitivity analysis showed no change in statistically significant levels, suggesting that the observed potential bias may not have substantially affected the summary measures. Finally, substantial heterogeneity was observed in the analysis of overall cancer, which was not surprising because of the variation in the effects of flavan-3-ols on cancer risk by cancer site. Therefore, subgrouping by cancer site reduced the apparent levels of heterogeneity.

The result from random-effect linearity meta-regression model suggested that there was no dose–response relationship between flavan-3-ols and cancer risk (data not shown). Furthermore, there are no data to suggest that long term use of high dosages of flavan-3-ols is detrimental to health. However, experimental studies based on physiologic dose levels are still needed to clarify an adequate range of exposures of flavan-3-ols.

To our knowledge, this is the first meta-analysis to comprehensively assess the effects of flavan-3-ols on the risk of a range of cancers. In the future, the findings should be confirmed by more well-designed cohort or intervention studies. And the underlying mechanisms of the different preventive effects of flavan-3-ols in various cancers remain to be elucidated.

## MATERIALS AND METHODS

### Search strategy

The PubMed, EMBASE and Cochrane databases were searched from their inception to January 2016 using the following terms in combination: flavan-3-ols (catechin, flavonoid), cancer (neoplasm, tumor, carcinoma) and epidemiology (case-control, cohort). In addition, the references of the retrieved articles were reviewed, and the searches were not limited by language.

### Study selection

An article was included in our meta-analysis if it (1) described a cohort or case-control study, (2) assessed flavan-3-ols (catechin) consumption via food intake, (3) provided a risk estimate (hazard ratio (HR), relative risk (RR), or odds ratio (OR)) and 95% CIs for cancer incidence, and (4) provided information on adjustment for confounding factors. Studies that evaluated urinary or plasma flavan-3-ols were excluded from this analysis. When an article could not be rejected with certainty based on the title and abstract, the full text was obtained, and inclusion was assessed independently by 2 reviewers (LL and YY).

### Data extraction and quality assessment

Data were extracted independently by 2 authors (LL and YY) using a common form, and differences were subsequently adjudicated. The standardized data extraction form collected the following: the study name (the first author's last name and the year of publication); the region; the study design; the type of controls for case–control studies (population-based or hospital-based controls); the sample size (cases and controls or cohort size); the follow-up time for cohort studies; the type of flavonoid intake; the comparison of flavan-3-ols intake; the type of cancer; the adjusted RR, OR or HR with 95% CIs for the highest compared with the lowest amount of flavan-3-ols consumed; and the most completely adjustment.

The assessment of study quality used the Newcastle-Ottawa Scale, which is a 9-star system including three dimensions: selection; comparability; and outcome (cohort studies) or exposure (case-control studies), depending on the study type [62]. An additional point was awarded when a study analyzed an energy-adjusted residual or nutrient-density model [7, 63], considering that the present meta-analysis assessed nutrient consumption and cancer risk. A study with≥7 points was considered to be a high-quality study using this 10-point system.

### Statistical methods

As the incidence of cancer was relatively low over the period of time studied, the most completely adjusted OR or HR (highest versus lowest flavan-3-ols intake) was considered to be an approximation of the RR. Studies were combined using the random-effects model, in which the effect measures are logRR weighted using the method of DerSimonian and Laird [64]. Statistical heterogeneity among studies was estimated using Q and I^2^ statistics [65]. To assess publication bias, funnel plots were constructed, and the methods of Begg's test (rank correlation) [66] and Egger's test (linear regression) [67] were used to test funnel plot asymmetry. If a potential bias was detected (P < 0.05), the trim and fill analysis was further used to examine the impact of possibly missing studies [68]. To assess whether the combined estimates could have been markedly affected by a single study, a sensitivity analysis was also conducted, in which each study was excluded one by one and the analysis was repeated based on the remaining studies. All data analyses were performed with Comprehensive Meta-Analysis Software, version v. 2.0 (CMA, Biostat, Englewood, NJ, USA).

## SUPPLEMENTARY MATERIALS TABLES




