# Supplementary material for: Flavan-3-ols consumption and cancer risk: a meta-analysis of epidemiologic studies
Source: Oncotarget. 2016 Sep 14;7(45):73573–92. doi: 10.18632/oncotarget.12017 (PMC5342000; doi:10.18632/oncotarget.12017)
Supplement: Supplementary file 1 [file oncotarget-07-73573-s001.pdf]

Flavan-3-ols consumption and cancer risk: A meta-analysis of epidemiologic studies

SUPPLEMENTARY TABLE

Supplementary Table S1: Flavonoids included in flavan-3-ols

See Supplementary File 1
