# Supplementary material for: Flavan-3-ols consumption and cancer risk: a meta-analysis of epidemiologic studies
Source: Oncotarget. 2016 Sep 14;7(45):73573–92. doi: 10.18632/oncotarget.12017 (PMC5342000; doi:10.18632/oncotarget.12017)
Supplement: Supplementary file 2 [file oncotarget-07-73573-s002.docx]

| **Supplementary** | **Table 1** |  |  |  |
| --- | --- | --- | --- | --- |
| Study | Name | Database | Flavonoids included | Food sources |
| Arts,2002 | Total catechin |  | (+)-Catechin, (+)-Gallocatechin, (-)-Epicatechin, (-)-Epigallocatechin, (-)-Epicatechin 3-gallate, (-)-Epigallocatechin 3-gallate | tea (59%), and apples and pears(26%) |
| Zamora-Ros,2014 | Flavan-3-ol monomers | The USDA databases for the flavonoid content of selected foods | (+)-Catechin, (+)-Gallocatechin, (-)-Epicatechin, (-)-Epigallocatechin, (-)-Epicatechin 3-gallate, (-)-Epigallocatechin 3-gallate | Tea (84%), fruits (6%), wine (3%), chocolates (2%) |
| Bosetti,2005 | Flavan-3-ols | The USDA databases for the flavonoid content of selected foods |  | red wine, grapes, and other  fruits |
| Fink,2007 | Flavan-3-ols | The USDA databases for the flavonoid content of selected foods |  |  |
| Peterson,2003 | Flavan-3-ols | The USDA databases for the flavonoid content of selected foods |  |  |
| Sanchez,2009 | Flavan-3-ols | The USDA databases for the flavonoid content of selected foods |  | Broad beans, Grapes, Blackberry, Milk chocolate, Red wine, Apple, Red plum |
| Touvier,2013 | catechins | The Phenol-Explorer database | (+)-Catechin, (+)-Gallocatechin, (-)-Epicatechin, (-)-Epigallocatechin, (-)-Epicatechin 3-gallate, (-)-Epigallocatechin 3-gallate | Tea, Chocolate, Alcoholic beverages, Fruit, Vegetables |
| Wang,2014 a | Flavan-3-ols | The USDA databases for the flavonoid content of selected foods |  | Nonherbal tea, Fresh apples or pears, Bananas, Blueberries (fresh, frozen, or canned), Applesauce, Red wine, Apple juice or cider, Muffins (regular) or biscuits, Strawberries (fresh, frozen, or canned) |
| Zamora-Ros,2013 a | Flavan-3-ol monomers | The USDA databases for the flavonoid content of selected foods | (+)-Catechin, (+)-Gallocatechin, (-)-Epicatechin, (-)-Epigallocatechin, (-)-Epicatechin 3-gallate, (-)-Epigallocatechin 3-gallate | Tea (86.3 %), apples and pears (2.9 %), wine (2.4 %), chocolates (1.8 %) |
| Kyle,2010 | Flavan-3-ols |  | (+)-Catechin, (+)-Gallocatechin, (-)-Epicatechin, (-)-Epigallocatechin, (-)-Epicatechin 3-gallate, (-)-Epigallocatechin 3-gallate |  |
| Mursu,2008 | Flavan-3-ols | The USDA databases for the flavonoid content of selected foods | (+)-Catechin, (+)-Gallocatechin, (-)-Epicatechin, (-)-Epigallocatechin, (-)-Epicatechin 3-gallate, (-)-Epigallocatechin 3-gallate, Theaﬂavin, Theaﬂavin-3-gallate, Theaﬂavin-3’-gallate, Theaﬂavin-3,3’-digallate, Thearubigins |  |
| Nimptsch,2016 | Flavan-3-ols |  | (+)-Catechin, (-)-Epicatechin, (-)-Epigallocatechin, (-)-Epicatechin 3-gallate, (-)-Epigallocatechin 3-gallate |  |
| Rossi,2006 | Flavan-3-ols | The USDA databases for the flavonoid content of selected foods |  |  |
| Simons,2009 | Total catechin |  | (+)-Catechin, (+)-Gallocatechin, (-)-Epicatechin, (-)-Epigallocatechin, (-)-Epicatechin 3-gallate, (-)-Epigallocatechin 3-gallate | broad beans, berries, grapes, black chocolate, tea and red wine |
| Theodoratou, 2007 | Flavan-3-ols |  | catechin, epicatechin, and gallates (epigallocatechin, epicatechin-3 gallate, epigallocatechin-3 gallate, and gallocatechin) | Tea (89.3%), apples (3.1%), red wine (2.1%) |
| Zamora-Ros, 2013 b | Flavan-3-ols | The USDA databases for the flavonoid content of selected foods | ﬂavan-3-ol monomers (catechin, epigallocatechin, epicatechin, epicatechin 3-gallate, epigallocatechin 3-gallate, gallocatechin, catechin 3-gallate) | Fruits (52.4 %), wine (23.7 %), chocolate products (6.5 %), tea (5.8 %) |
| Bobe,2009 | Flavan-3-ols | The USDA databases for the flavonoid content of selected foods | (+)-Catechin, (+)-Gallocatechin, (-)-Epicatechin, (-)-Epigallocatechin, (-)-Epicatechin 3-gallate, (-)-Epigallocatechin 3-gallate | Iced tea, hot tea, beer, apples/pears, red wine |
| Petrick,2015 | Flavan-3-ols | The USDA databases for the flavonoid content of selected foods | (+)-Catechin, (+)-Gallocatechin, (-)-Epicatechin, (-)-Epigallocatechin, (-)-Epicatechin 3-gallate, (-)-Epigallocatechin 3-gallate, Theaﬂavin, Theaﬂavin-3-gallate, Theaﬂavin-3’-gallate, Theaﬂavin-3,3’-digallate, Thearubigins | Black tea (83.7%), beer (3.2%), green tea (3.1%), wine (2.0%), apples (1.9%), bananas (1.4%) |
| Rossi,2007 a | Flavan-3-ols | The USDA databases for the flavonoid content of selected foods |  | Tea, wine and fruit |
| Vermeulen, 2013 | Flavan-3-ol monomers | The USDA databases for the flavonoid content of selected foods | (+)-Catechin, (+)-Gallocatechin, (-)-Epicatechin, (-)-Epigallocatechin, (-)-Epicatechin 3-gallate, (-)-Epigallocatechin 3-gallate |  |
| Lagiou,2004 a | Flavan-3-ols | The USDA databases for the flavonoid content of selected foods |  |  |
| Rossi,2010 | Flavan-3-ols | The USDA databases for the flavonoid content of selected foods |  |  |
| Woo,2014 | Flavan-3-ols | The USDA databases for the flavonoid content of selected foods | catechin, epigallocatechin, epicatechin, epicatechin 3-gallate, epigallocatechin 3-gallate, gallocatechin, catechin 3-gallate, theaflavin, thearubigins, theaflavin-3,3-digallate, theaflavin-3-gallate, and theaflavin-3′-gallate |  |
| Zamora-Ros, 2012 | Flavan-3-ol monomers | The USDA databases for the flavonoid content of selected foods | ﬂavan-3-ol monomers (catechin, epigallocatechin, epicatechin, epicatechin 3-gallate, epigallocatechin 3-gallate, gallocatechin, catechin 3-gallate) |  |
| Rossi, 2007 b | Flavan-3-ols | The USDA databases for the flavonoid content of selected foods |  |  |
| Garavello, 2007 | Flavan-3-ols | The USDA databases for the flavonoid content of selected foods |  | Tea, wine and fruits |
| Lagiou,2008 | Flavan-3-ols | The USDA databases for the flavonoid content of selected foods |  | Various fruits including apples, apricots, and cherries |
| Zamora-Ros, 2013 c | Flavan-3-ol monomers | The USDA databases for the flavonoid content of selected foods | (+)-Catechin, (+)-Gallocatechin, (-)-Epicatechin, (-)-Epigallocatechin, (-)-Epicatechin 3-gallate, (-)-Epigallocatechin 3-gallate | Tea (83.9%), fruits (5.9%), wines (3.4%), chocolate products (1.8%) |
| Arts, 2001 | Total catechin |  | (+)-Catechin, (+)-Gallocatechin, (-)-Epicatechin, (-)-Epigallocatechin, (-)-Epicatechin 3-gallate, (-)-Epigallocatechin 3-gallate | Tea (88.6%), Apple (8%), Chocolate (2.9%) |
| Christensen, 2012 | Flavan-3-ols | The USDA databases for the flavonoid content of selected foods |  | Black tea (92%), Apples/pears (2%), Beer (2%) |
| Cui,2008 | Epicatechin  Catechin |  | Catechin and Epicatechin |  |
| Cutler,2008 | Flavan-3-ols | The USDA databases for the flavonoid content of selected foods |  |  |
| Lagiou,2004 b | Flavan-3-ols | The USDA databases for the flavonoid content of selected foods |  |  |
| Rossi,2008 | Flavan-3-ols | The USDA databases for the flavonoid content of selected foods |  | Tea (50%), Apples and pears (20%), Wine (15%) |
| Arem,2013 | Flavan-3-ols | The USDA databases for the flavonoid content of selected foods |  |  |
| Bobe,2008 | Flavan-3-ols |  | sum of catechin and epicatechin | tea, apples, and red wine |
| Rossi,2012 | Flavanols | The USDA databases for the flavonoid content of selected foods |  | tea, apples, pears and wine |
| Bosetti,2006 | Flavan-3-ols | The USDA databases for the flavonoid content of selected foods |  | tea, wine, and fruits |
| Geybels, 2013 | Total catechin |  | (+)-Catechin, (+)-Gallocatechin, (-)-Epicatechin, (-)-Epigallocatechin, (-)-Epicatechin 3-gallate, (-)-Epigallocatechin 3-gallate | Black tea, Apples/pears, Chocolate, Broad beans |
| Wang,2014 b | Flavan-3-ols | The USDA databases for the flavonoid content of selected foods |  | Nonherbal tea, Bananas, Fresh apples or pears, Beer, Red wine, Blueberries (fresh, frozen, or canned), Applesauce, Herbal or decaffeinated tea, Apple juice or cider, Nonchocolate sweets or baked goods |
| Bosetti,2007 | Flavan-3-ols | The USDA databases for the flavonoid content of selected foods |  | Tea, wine, and fruits |
| Xiao,2014 | Flavan-3-ols | The USDA databases for the flavonoid content of selected foods |  |  |
| Rossi,2013 | Flavanols | The USDA databases for the flavonoid content of selected foods |  | Tea, apples, pears and wine |
